# Acoustic and thermal insulation properties of rubberhemp shive composite bonded with regenerated polyurethane resin

**DOI:** 10.1038/s41598-026-35411-x

**Published:** 2026-01-08

**Authors:** Tomas Astrauskas, Giedrius Balčiūnas, Jolita Bradulienė, Robert Ružickij, Andrej Naimušin, Tomas Januševičius

**Affiliations:** 1 Research Institute of Environmental Protection, Vilnius Gediminas Technical University, Saulėtekio al. 11, Vilnius, Lithuania; 2 Laboratory of Thermal Insulating Materials and Acoustics, Vilnius Gediminas Technical University, Linkmenų g. 28, Vilnius, Lithuania

**Keywords:** Sound absorption, Thermal conductivity, Rubber granule, Hemp shive, Composite materials, Recycling, Energy science and technology, Engineering, Materials science

## Abstract

Studies on recycled materials for sound absorption and thermal insulation applications has emerged in recent years. This paper investigates the composite material made of the rubber granules and hemp shives. The rubber granule was gained from tyre recycling factory, and hemp shives which is still considered as waste in hemp fibre production. This paper analyses the hemp shives influence on acoustic and non-acoustic properties of rubber granule and hemp shive (RGHS) composites bonded with regenerated polyurethane resin. Tested composite material samples varied in rubber grain (RG) size (0.5–2 mm; 24 mm; 4–6 mm) and hemp shives (HS) (avg. length 7 mm; avg. width 2.2 mm). In this study the HS quantity was controlled and increased by ratio from 0 to 1:1. Samples of four thicknesses (10, 20, 30, 50 mm) were tested to find the HS influence on sound absorption coefficient. For non-acoustic parameters estimation gas pycnometry and inverse characterisation method according to Johnson–Champoux–Allard (JCA) model was used. The results showed that some of the parameters were influenced by change of hemp shive quantity in the composite panel. HS content in the composite increment influenced the airflow resistivity decrement by 50–57%, bulk density decrement by 21–28%, porosity increment by 12–17%. The acoustic sound absorption performance was tested using impedance tube transfer function method (ISO 10354–2). The peak sound absorption coefficient varied from 0.60–0.97 depending on the sample thickness and configuration. The correlation between hemp shive quantity and sound absorption was not significant. The test of thermal conductivity according to EN 12664 showed that minimum value of the RGHS panels of thermal conductivity coefficient was 0.07 W/m·K. Such results indicate that RGHS could be developed as multi-purpose material for sound absorption and thermal insulation applications. The aim of this paper was to incorporate HS into rubber granule panels and to investigate its influence on the acoustic and non-acoustic properties on the RGHS composite panels.

## Introduction

Many countries in the world are facing many waste management problems. Due to the generation of solid industrial and municipal waste, the waste management becomes one of the main chalenges in the mitigation of climate change^1^. Current waste management solutions are not efficient enough and waste recycling technologies are currently being developed. Therefore, there is a gap in knowledge on how to utilise various wastes for the production of new recycled products. The construction sector is known to use different waste materials as fillers to improve the properties of primary materials. In particular, a lot of research has been carried out to reduce noise pollution: new road surfaces are being developed from nanomaterials, adding bio-based additives^2^, mixing tyre rubber into the asphalt^3^, building construction elements are developed from recycled polymers, natural fibres and biological resins^4–7^. Some waste recycling technologies consume a lot of energy. As an example, to reduce energy consumption to recycle waste rubber from tyres, it is proposed to devulcanize them using a biotechnological method with microorganisms and enzymes^8^.

Recycling tyre rubber helps mitigate the environmental hazards associated with tyre waste, such as soil and water contamination, and air pollution from burning tyres. The main component in the tyres is carbon isoprene (C_5_H_8_) which is significant organic volatile compound. Under combustion condition this component form the pollutants such as CO and various hydrocarbons, which are significant in atmospheric chemistry and pollutant formation. In addition, recycling reduces the volume of waste tyres in landfills, which are known to leach toxic chemicals and pose fire hazards^9,10^. By recycling tyres, the demand for new raw materials is reduced, conserving natural resources and reducing greenhouse gas emissions. Recycled rubber is used in cement mixtures, road construction, and geotechnical work, contributing to sustainable development and environmental protection. PU is a polymer composed of hard and soft segments chemically linked with covalent bonds^11^. These properties can be used to achieve various properties of materials. Polyurethane adhesives are commonly used to bond rubber particles^12^. Studies show that PU and rubber composites show particular interest of research for sound absorption applications^13^. The different rubber grain size composition panels with gypsum boards resulted in variation of sound transmission loss from 61.9 to 67.4 dB. Similarly, insertion loss values ranged from 20 to 35 dB^14^. The sound absorption peak of the tyre rubber granule panels bonded with the polyurethane resin reached 0.90, the peak frequency depends on the thickness of the sample^15^. Tyre rubber is sometimes used in asphalt or cement composites. Studies have shown that the rubber granule in cement composites tend to increase the sound absorption coefficient values^16,17^.

The research of natural materials for sound absorption applications is relevant as well. The studies show that sound absorption coefficient of the multilayer coir, jute and bamboo composites reached up to 0.90^18^. The addition of jute fibre treated with amino silicone to the propylene composite material increased the average sound absorption coefficient by 120%^19^. The biocomposite made of layers of jute and aloe showed a sound absorption coefficient of 0.47 (thickness 3.4 mm) at 3 kHz and a transmission loss of 19.84 dB^20^. Wood-based fibre foams showed nearly perfect sound absorption at 1000 Hz when sample thickness was 6 cm^21^. Due to its versatility, low cultivation costs, short production cycle and low CO_2_ emissions hemp fibre become increasingly popular material^22,23^. The hemp processing generates the by-products called hemp shives (HS). The composition of hemp shives is around 75% hemp stem which is wooden part and 25% of fiber^24^. Studies of other authors suggest that hemp shives are porous material which ultimately could mean that such material is good for sound absorption applications^25^. The studies show hemp shives often used in various cement composites (hempcrete). The sound absorption coefficient of hempcrete composites could reach up to 0.9 depending on composition of the material^26^.

Hemp shive composites showed thermal conductivity values that generally range from 0.049 to 0.068 W/m·K under dry to moderately humid conditions, making them competitive with other biobased materials^27–30^. Key factors influencing thermal conductivity include density, moisture content (15–20% increase from dry to 90% RH), particle size (larger particles reduce thermal conductivity by up to 7.6%), and binder type and ratio (higher binder content increases thermal conductivity by ~ 20%)^31–34^. In contrast, composite materials incorporating waste tyre rubber generally exhibit higher thermal conductivity compared with hemp shive composites, with values ranging from 0.05 to 0.3 W/m·K depending on matrix type, filler content, and processing, making them somewhat effective thermal insulators^35–37^. Furthermore, increasing the rubber content in the composite generally reduces both thermal conductivity and density by up to 69%, while particle size continues to influence both thermal and mechanical properties^38–40^.

In general, the various studies and their quantity show that the noise pollution mitigation using waste materials is a relevant topic. The knowledge about hemp shive and tyre rubber granule panels bonded with polyurethane resin is still limited. Limited number of studies separately analysed rubber or hemp panels acoustic and thermal insulation. Such composite material where hemp shive and rubber granule panels acoustic and thermal properties are still unknown. In this paper we propose the new specification of rubber granule composite panel mixed with hemp shives and bonded with polyurethane resin for sound absorption and thermal insulation applications. Although hemps are considered as environmentally friendly material, hemp shives are the only waste that is produced in the sector. This study incorporates hemp shives into rubber-based panels and systematically evaluate their influence on both acoustic and thermal properties. This approach not only valorises two waste streams but also proposes a scalable solution aligned with circular economy principles. In this paper, we present the study in which the aim was to incorporate hemp shives into rubber granule panels and to investigate its influence on the acoustic and non-acoustic properties on the composite material.

The paper is organised as follows: In the *Materials and methods* section, the methods of testing and materials which were used are presented. The *Results and Discussion Section* presents and discusses the main results of the study. In the *Conclusions* section, the main conclusions of the study are presented.

## Materials and methods

**Fig. 1:**
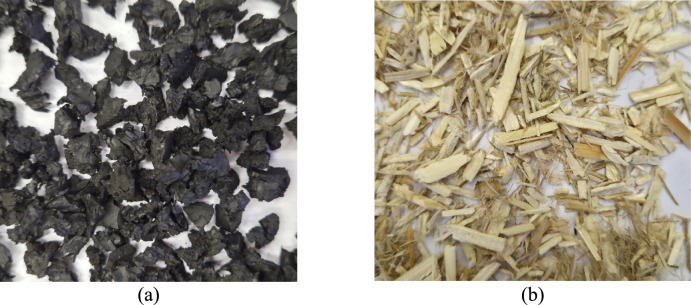
Raw materials used in the study, **(a)** rubber granules, **(b)** hemp shives.

In this section, the methods and materials that were used in this study are presented. In this study, rubber granule and hemp shive composite panels are analysed. Recycled rubber granules obtained from end-of-life tyres were used to produce panels (Fig. 1.(a)). The hemp shive is the waste material that is generated during the processing of hemp fibre (Fig. 1 (b)).

Rubber granules and hemp shives were provided by the Lithuanian rubber recycling company.

### Method of sample preparation

Rubber granules was separated according to a grain size. Rubber granules and hemp shives for sample production was taken using envelope sampling method. A total of 18 sample compositions of rubber granules and hemp shives (RGHS) were produced for this study, and samples of each composition were manufactured with different thicknesses. The thicknesses of the samples gradually varied from 10 to 50 mm. Table No. 1 shows the compositions and thicknesses of the samples. The bulk density of the hemp shives used in this study was 92.7 kg/m^3^.

**Fig. 2:**
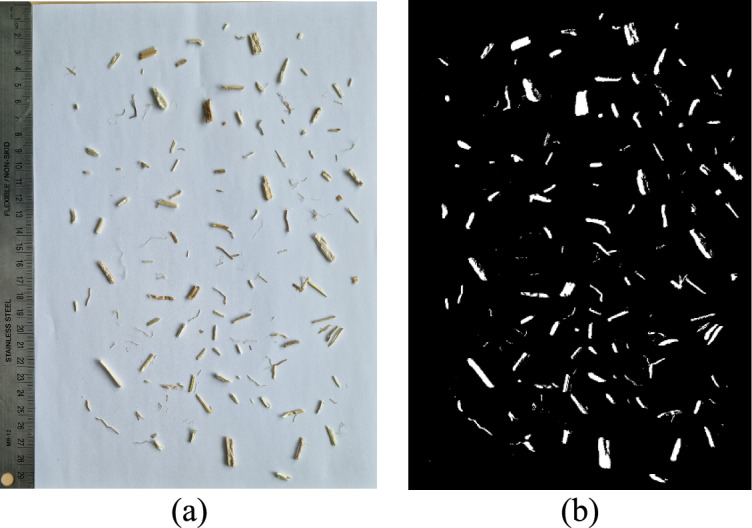
Image used for particle size evaluation **(a)** raw image, **(b)** processed image.

The hemp shives were taken from the hemp processing factory in Lithuania. Since hemp shives are elongated particles, it is nearly impossible using the traditional sieving method. To determine the particle size of the hemp shives, the image processing software Fiji was used^41^. Two photos (Fig. 2) were taken with a smartphone (Samsung Galaxy S21 Ultra) camera in resolution of 108 MP. In total 309 hemp shive particles were measured.

Polyurethane resin was used as a binding material in all samples. In sample production the a 2-component polyurethane (2 K) system was used. The components used for Polyurethane resin production was polyol and Methylene diphenyl diisocyanate (pMDI). The ratio between components was 3:1. The polyol and pMDI were granted by local company of Lithuania, JSC “Provectus Redivivus”. The model of polyol resin is Ecopol KV. The main properties of polyol are presented in Table 1. pMDI consists of monomeric methylene diphenyl diisocyanate (MDI) and higher oligomeric homologues compared to MDI. It is a translucent brown mixture containing approximately 50% monomeric MDI and higher molecular weight multiring species.

**Table 1:** The main properties of used polyol in this study.

Density g/ml	Viscosity (mPa∙s)	Acid number (mg KOH/g)	Hydroxyl Number (mg KOH/g)	Hydroxyl equivalent %
1.1 ± 0.1	455 ± 20 (25 °C)	7.2 ± 0.5	45 ± 7	1.4 ± 0.2

This method does not use any solvents; therefore, it is more environmentally friendly. Firstly, rubber granules were manually mixed with hemp shives. Then polyol and pMDI was mixed separately until reaction formed even mass and then poured into mould with RGHS mixture. To reduce bubble formation due to the viscosity of the polyurethane resin, the mixture was manually compacted and gently vibrated using dental vibrator plate after pouring into the moulds to release trapped air. Fully mixed composite material left to dry and harden for 24 h at room temperature. The detailed composition of RGHS panels presented in Table 2.

**Table 2:** Composition of RGHS panels.

No	Particle size of rubber, (mm)	Thickness, (mm)	Average length of hemp shives, (mm)	Average width of hemp shives, (mm)	Ratio of rubber granule to hemp shives	Percentage of Polyurethane resin, (wt%)
1	0.5–2	10 ± 2; 20 ± 3; 30 ± 3; 50 ± 3	7.0 ± 4.5	2.2 ± 1.6	1:0	25 ± 0.3
2					9:1	35 ± 0.3
3					4:1	40 ± 0.8
4					2.3:1	45 ± 1.8
5					1.5:1	55 ± 1.7
6					1:1	57 ± 0.7
7	2–4	10 ± 2; 20 ± 3; 30 ± 3; 50 ± 3			1:0	25 ± 1.9
8					9:1	35 ± 1.6
9					4:1	40 ± 0.7
10					2.3:1	45 ± 0.7
11					1.5:1	55 ± 0.8
12					1:1	57 ± 0.1
13	4–6	10 ± 2; 20 ± 3; 30 ± 3; 50 ± 3			1:0	25 ± 4.6
14					9:1	35 ± 3.9
15					4:1	40 ± 2.5
16					2.3:1	45 ± 0.7
17					1.5:1	55 ± 0.2
18					1:1	57 ± 0.8

The amount of binder increased with hemp shive quantity due to hydrophilicity of the hemp shives. Hemp shives have an average accessible porosity of around 75–80% and can absorb water up to 300–400% of their own weight. This extreme absorbency is due to the hydrophilic structure – its open pores readily accept liquids^42^. In contrast, recycled rubber from tyres is a dense, material with low porosity; crumb rubber is hydrophobic and absorbs virtually no water or liquid binder^43^.

Due to this difference, polyurethane resin is absorbed into hemp shives’ pores, whereas it stays on the surface of rubber granules. Due to this phenomenon, more resin must be added to saturate the shives and still have enough left to bond all the particles together.

**Fig. 3:**
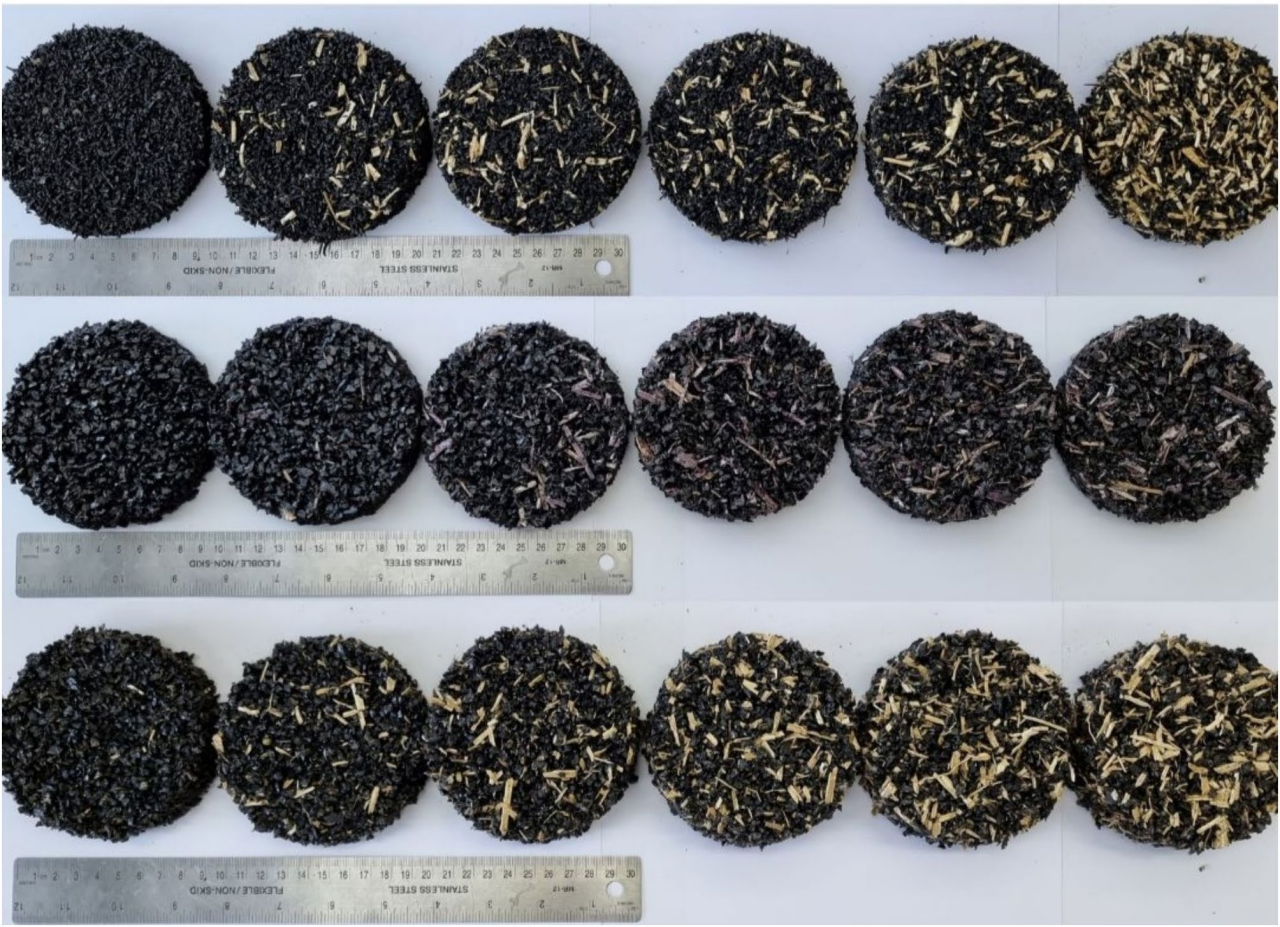
RGHS panel samples for 100 mm diameter impedance tube.

The samples prepared for impedance tube measurements are presented in Fig. 3. The size of the formed panels was 300 × 300 mm and then cut according to the needs for each testing method. A total of 3 samples were produced for each composition. Each composition panel was produced of different thickness (Table 2). In total, 216 samples were produced for this study.

### Method of determination of bulk and true density and estimation of porosity of RGHS samples

**Fig. 4:**
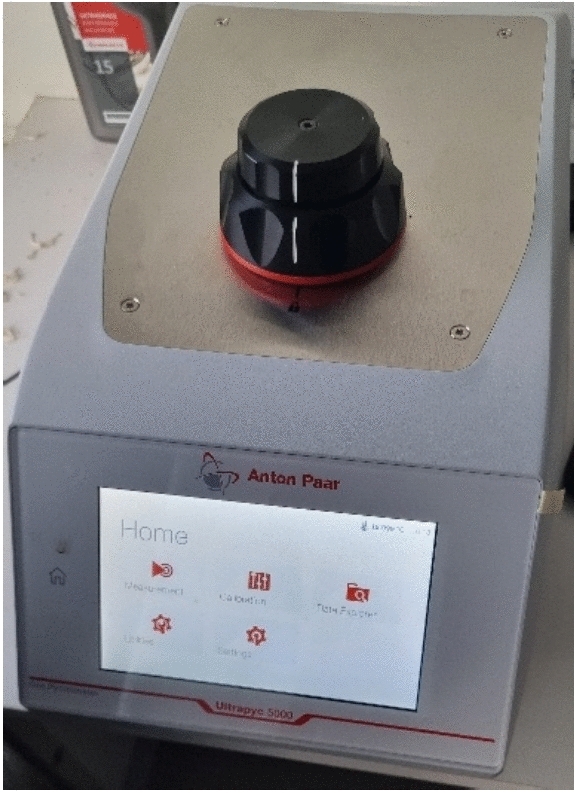
Gas pycnometer.

The true density of RGHS was determined using an automated gas pycnometer (Fig. 4). The method relies on the Archimedes principle of fluid displacement and Boyle’s law of gas expansion to measure the true volume and density of the material. The displaced fluid in gas pycnometry is an inert gas that can penetrate all but the finest pores to assure maximum accuracy. The nitrogen gas was used for pycnometric measurements.

For gas pycnometric measurements, a sample of known mass is put into a sample chamber of known volume, which is then sealed and pressurised with inert gas to a pre-defined value. The mass (*m*) of the samples was measured with analytical scales with accuracy of 0.1∙10^–6^ kg. After the pressure in the sample chamber has stabilised, a valve that connects the sample chamber to a reference chamber (whose volume is also known) is opened.

The gas expands into this new volume. Instrument use the resulting pressure readings to calculate the volume of the sample using the following Eq. 1:

1 $$\begin{array}{*{20}c} {V_{s} = V_{c} - \frac{{V_{R} }}{{\left( {\frac{{P_{i} }}{{P_{f} }}} \right) - 1}} } \\ \end{array}$$

Where V–true volume of the sample, V_c_–volume of the empty chamber; V_R_–volume of the empty chamber; P_i_–initial pressure; P_f_–final pressure after expansion. After measurement true density is calculated according to Eq. 2:

2 $$\begin{array}{*{20}c} {\rho_{t} = \frac{m}{{V_{s} }}} \\ \end{array}$$

where: ρ_t_–true density; m–mass of the sample. Eventually the open porosity (φ) of the RGHS was calculated^44^ according to Eq. 3:

3 $$\begin{array}{*{20}c} {\varphi = \frac{{\rho_{t} - \rho_{b} }}{{\rho_{t} }}} \\ \end{array}$$

where: φ – porosity of the material; ρ_b_ – bulk density. The porosity value was used for sound absorption coefficient prediction of RGHS.

### Method of determination of normal incidence sound absorption coefficient and high frequency limit dynamic density

**Fig. 5:**
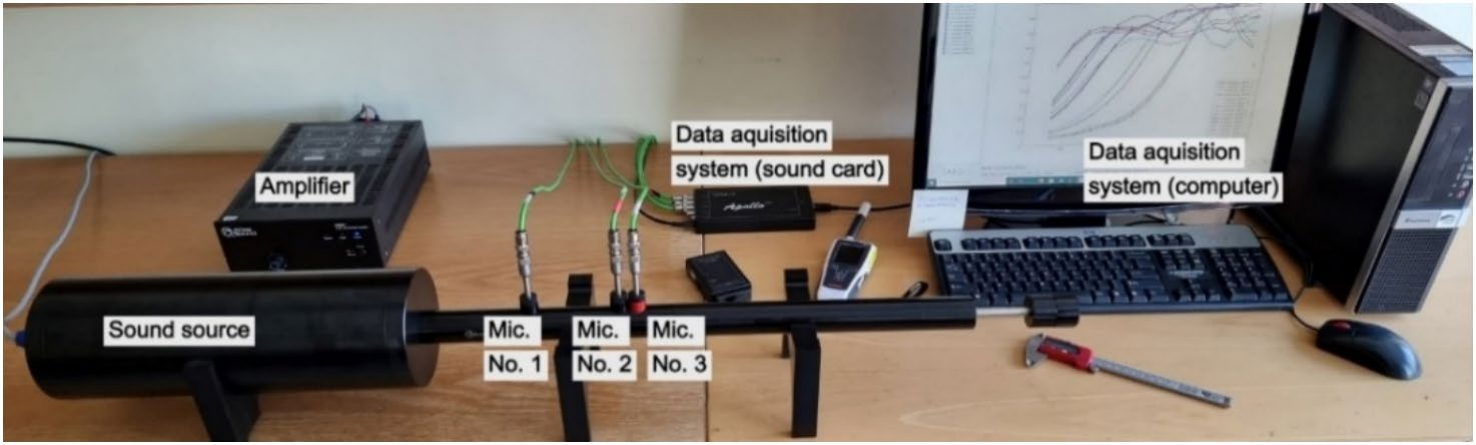
The impedance tube configuration for sound absorption measurements.

To obtain the sound absorption coefficient (SAC) (α) of RGHS, the ISO 10534–2 standard method was used^45^. Such method was chosen due to the requirements for sample size compared with methods which can be performed in reverberant rooms. Two impedance tubes used in the study was manufactured by Gesellschaft für Akustikforschung Dresden GmbH, product AcoustiTube. The three-microphone technique was implemented to get less noisy data. Two tubes allowed to measure SAC in wider frequency range. For lower frequency range (50–2000 Hz) measurements – 100 mm inner diameter tube was used, for higher frequency range (150–6600 Hz) – 30 mm inner diameter tube. The experimental setup with a 30 mm inner diameter tube is shown in Fig. 5. The total measured frequency range was 50–6600 Hz. The white noise signal was used in this study, because it contains equal energy across frequency range allowing simultaneous measurement of acoustic properties of materials without performing multiple single-frequency tests. This approach significantly reduces measurement time while maintaining accuracy.

The transfer function method was used with the three-microphone technique.

The transfer function ${H}_{13}$ between microphone positions is calculated as the pressure ratio between pressures measured by both microphones (Eq. 4). The transfer function for incident wave ${H}_{I}$ and transfer function for reflected wave ${H}_{R}$ calculated according to Eqs. (5) and (6) ^45^:

4 $$\begin{array}{*{20}c} {H_{13} = \frac{{p_{3} }}{{p_{1} }} , H_{23} = \frac{{p_{3} }}{{p_{2} }}} \\ \end{array}$$

5 $$\begin{array}{*{20}c} {H_{{I\left( {mic1 - 3} \right)}} = \frac{{p_{3I} }}{{p_{1I} }} = e^{{ - jk_{0} \left( {x_{12} + x_{23} } \right)}} ; H_{{I\left( {mic2 - 3} \right)}} = \frac{{p_{3I} }}{{p2_{I} }} = e^{{ - jk_{0} \left( {x_{23} } \right)}} } \\ \end{array}$$

6 $$\begin{array}{*{20}c} {H_{{R\left( {mic1 - 3} \right)}} = \frac{{p_{3R} }}{{p_{1R} }} = e^{{jk_{0} \left( {x_{12} + x_{23} } \right)}} ;H_{{R\left( {mic2 - 3} \right)}} = \frac{{p_{3R} }}{{p_{2R} }} = e^{{jk_{0} \left( {x_{23} } \right)}} } \\ \end{array}$$

From Eq. (3), (4), and (5) the reflection coefficient of the sample can be calculated as:

7 $$\begin{array}{*{20}c} {R_{{\left( {mic1 - 3} \right)}} = \frac{{H_{13} - H_{{I\left( {mic1 - 3} \right)}} }}{{H_{{R\left( {mic1 - 3} \right)}} - H_{12} }}e^{{2jk_{0} \left( {X_{12} + X_{23} + X_{3s} } \right)}} ;R_{{\left( {mic2 - 3} \right)}} = \frac{{H_{23} - H_{{I\left( {mic2 - 3} \right)}} }}{{H_{{R\left( {mic2 - 3} \right)}} - H_{13} }}e^{{2jk_{0} \left( {X_{23} + X_{3s} } \right)}} } \\ \end{array}$$

where: R is the reflection coefficient of the sample, k_0_ is the wavenumber in air.

Finally, the sound absorption coefficient *α* is calculated using the following expression:

8 $$\begin{array}{*{20}c} {\alpha = 1 - \left| R \right|^{2} } \\ \end{array}$$

For engineering applications, sound spectra are often presented as one-third octave frequency bands rather than narrow bands. This frequency representation is linked to the perception of sound by the human ear and allows compression of the amount of information. In this paper, the results are presented in 1/3 octave to show relevant information while focussing on the application possibilities of the RGHS panels. Each composition consists of three samples for each impedance tube, and 100 averages were made for each measurement. In total, 216 separate samples were tested to obtain the SAC data.

**Fig. 6:**
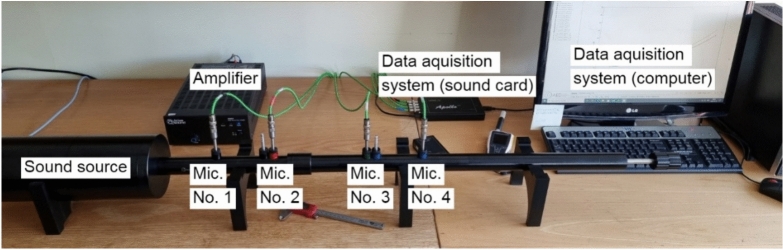
Transmission tube setup.

To determine effective density (${\rho }_{e}$) and characteristic impedance of the material *(Z*_*c*_*)*, a 30mm inner diameter transmission tube was used. The measuring system in total contains four microphones. The measurement system is presented in Fig. 6.

The transfer matrix for such measurement system is as^46^:

9 $$\begin{array}{*{20}c} {T = \left[ {\begin{array}{*{20}c} {T_{11} } & {T_{12} } \\ {T_{21} } & {T_{22} } \\ \end{array} } \right]} \\ \end{array}$$

To evaluate JCA (Johnson-Champoux-Allard) model parameters additional acoustic parameters were extracted to calculate non-acoustic parameters. The characteristic impedance of the material was calculated using the transmission tube according to ASTM E2611 standard. The propagation of the wave number in material *k’* is determined using Eq. 10^46^:

10 $$\begin{array}{*{20}c} {k^{\prime} = \frac{1}{d}\cos^{ - 1} T_{11} } \\ \end{array}$$

Characteristic impedance *Z*_*c*_ in material calculated according to Eq. 11:

11 $$\begin{array}{*{20}c} {Z_{c} = \sqrt {\frac{{T_{12} }}{{T_{21} }}} } \\ \end{array}$$

After measurement using the data, it is possible to determine the effective density *ρ*_*eff*_ and the effective bulk modulus *K*_*eff*_ of the material according to a equation system No. 12^47^:

12 $$\begin{array}{*{20}c} {\left\{ {\begin{array}{*{20}c} {Z_{c} \left( \omega \right) = \sqrt {\rho_{eff} \left( \omega \right)K_{eff} \left( \omega \right)} } \\ {k^{\prime}\left( \omega \right) = \sqrt {\frac{{\rho_{eff} \left( \omega \right)}}{{K_{eff} \left( \omega \right)}}} } \\ \end{array} } \right.} \\ \end{array}$$

### Method of determination of JCA model parameters

In this section, the method of determination of Johnson-Champoux-Allard (JCA) model parameters is presented. The JCA model is widely used to characterise the acoustic properties of porous materials, particularly in the context of sound absorption. The parameters of the JCA model, such as porosity, flow resistivity, tortuosity, and characteristic lengths, are essential to accurately predicting the sound absorption properties of materials^48,49^. These parameters directly influence the model’s ability to predict how sound waves interact with the material, which is critical for designing effective sound-absorbing materials.

As previously written, porosity (*φ*) was measured directly. The other parameters were obtained by using the inverse characterisation from impedance tube measurements.

According to other authors and standard ISO 9053 the air flow resistivity of the material can be determined using impedance tube data^50–52^.

13 $$\begin{array}{*{20}c} {\sigma = \mathop {\lim }\limits_{\omega \to 0} - \omega {\mathrm{Im}} \left( {\rho_{e} } \right)} \\ \end{array}$$

The high frequency tortuosity (${\tau }_{\infty }$) of the material calculated according to Eq. 14^53,54^. In this formula, asymptotic values of the characteristic impedance of the real part *Re (Z*_*c*_*)* were used:

14 $$\begin{array}{*{20}c} {\tau_{\infty } = \left. {{\mathrm{Re}} \left( {\frac{{Z_{c} }}{{\rho_{0} c_{0} }}} \right)^{2} } \right|_{\omega \to \infty } } \\ \end{array}$$

The viscous characteristic length (*Λ*) according to^55^ has a relationship with other non-acoustic parameters ($\sigma ,\varphi ,{\alpha }_{\infty }$) and could be written as Eq. 15:

15 $$\begin{array}{*{20}c} {\Lambda = c\sqrt {\left( {\frac{{8\alpha_{\infty } \eta }}{\sigma \varphi }} \right)} } \\ \end{array}$$

The viscous thermal length $\Lambda {\prime}$ was calculated according to Eq. 16, even though the proposed formulation according to^47^ is best suited when the porosity of the material is up to 1. The curve fitting method showed a good enough correlation to the proposed RGHS samples.

16 $$\begin{array}{*{20}c} {\Lambda^{\prime} = 2\Lambda } \\ \end{array}$$

All these parameters were fitted into the JCA model, and the accuracy of the model was determined for each sample composition.

### Method of sound absorption average calculation

To summarise and compare the sound absorption average (SAA) was calculated. SAA is a single numerical value that represents the overall sound absorption performance of a material over a range of frequencies. SAA is calculated by measuring the sound absorption coefficients and averaging them at twelve 1/3 octave frequencies ranging from 200 to 2500 Hz. The calculation of SAA is done according to Eq. 17.

17 $$\begin{array}{*{20}c} {SAA = \frac{1}{n}\mathop \sum \limits_{i = 1}^{i} \alpha \left( {f_{i} } \right)} \\ \end{array}$$

where: $\alpha ({f}_{i})$ is the sound absorption coefficient at the *i*^th^ frequency, *n* is the total number of frequencies measured.

After the calculation of SAA, the comparative analysis is done to define the performance of the RGHS samples.

### Method of determination of thermal conductivity coefficient

Thermal conductivity is measured in accordance with EN 12664 "Thermal performance of building materials and products—Determination of thermal resistance by means of guarded hot plate and heat flow meter methods—Dry and moist products of medium and low thermal resistance". The measurement apparatus operates according to the principles set out in "ISO 8301 Thermal insulation — Determination of steady-state thermal resistance and related properties — Heat flow meter apparatus". In this method, heat flow sensors are attached to the surface of the specimen. The sensors measure the heat flow through the specimen, allowing for the calculation of thermal resistance on the heat transfer data.

**Fig. 7:**
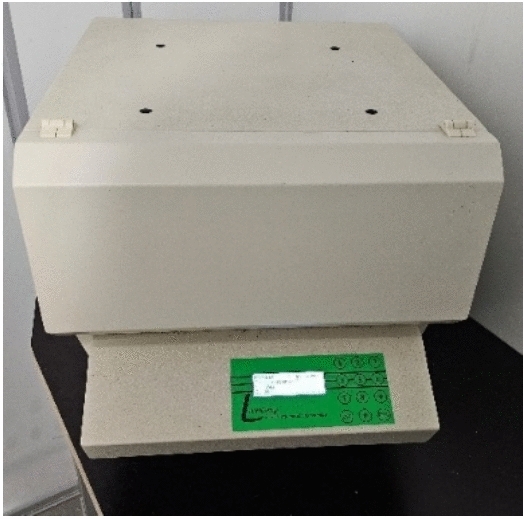
The equipment for thermal conductivity characterization.

Thermal conductivity is determined by measuring the heat flow, the thickness of the sample, and the temperature difference between the samples. Thermal conductivity was measured with a symmetrical configuration of the LaserComp FOX 304 horizontal heat flow measuring device with active protection of the edges of the sample in the direction of heat flow from the bottom to the top. Sample dimensions 300 × 300 mm, sample surface temperature difference 20 °C, average test temperature 10 °C. Measurement equipment is presented in Fig. 7.

### Method of scanning electron microscopy

**Fig. 8:**
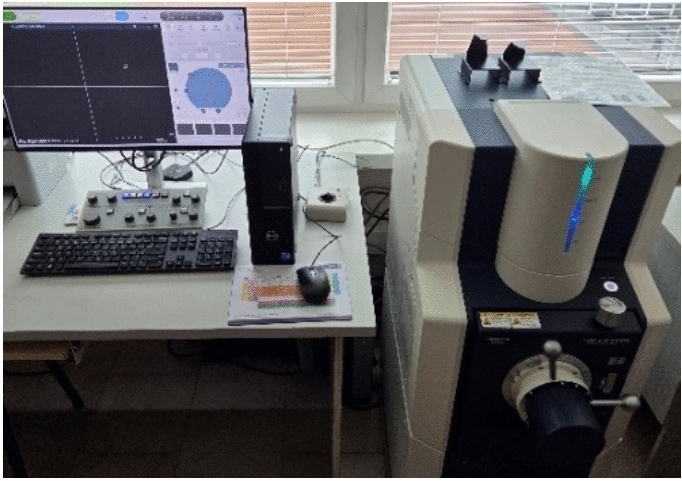
Scanning electron microscope used in the study.

The microstructural parameters of the RGHS composite samples were examined using a Flex SEM 1000 scanning electron microscope (SEM) (Fig. 8).

The samples were cut into small fragments (30 × 30 mm) to expose the fractured cross sections and the surface morphology. Before imaging, the specimens were cleaned with compressed air to remove loose particles and then mounted on an aluminium stub (80 mm diameter) using conductive carbon stickers. SEM observations were carried out under high vacuum conditions at an accelerating voltage of 1–15 kV using the secondary electron (SE) detection mode to capture detailed surface topography. The micrographs obtained were analysed to assess the bonding quality between rubber granules, hemp shives, and polyurethane resin matrix, as well as to identify the presence of gaps, microcracks, or delamination in the interfacial regions. Multiple magnifications were used to characterise both the general structure and fine details of the polymer–rubber and hemp particle interface.

## Results

### Non-acoustic parameters of RGHS panels

**Fig. 9:**
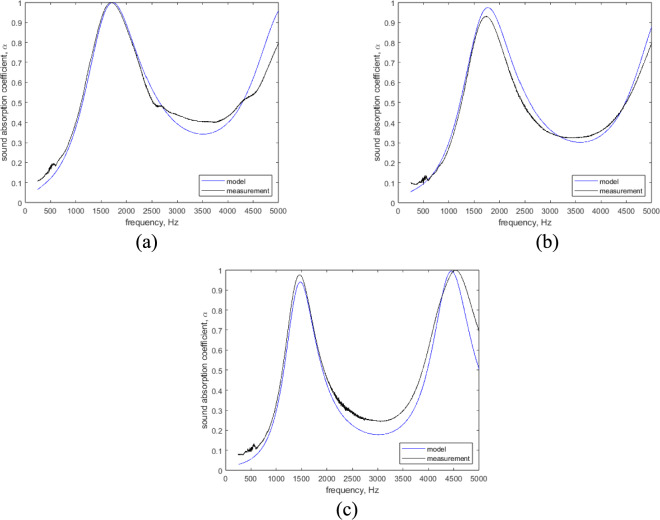
Validation of the model. **(a)** 0,52 mm rubber granule sample, **(b)**24 m rubber granule sample, (c) 46 mm rubber granule sample.

The true density and porosity values were determined using the gas pycnometry method described in Sect. 2.2. Other non-acoustic parameters determined by using a JCA model inversion method described in Sect. 2.3. The model was validated by comparing predicted sound absorption coefficients with measured sound absorption. The model showed good correlation with measured sound absorption. The fitting of the models presented in Fig. 9. The model demonstrates good agreement with the experimental measurements across the entire frequency range, accurately reproducing the main absorption peaks and overall trend.

The values of densities, high-frequency tortuosity, porosity, air flow resistivity, viscous and characteristic thermal lengths are presented in .

Table 3. In the table, it is shown that both densities (true and bulk) and air flow resistivity decreases with the addition of hemp shives in the composite. Such results occur because the hemp shives had a lower mass compared to the rubber granules. The bulk density was 92.7 kg/m^3^ while a previous study^56^ was shown that the bulk density rubber granules were 315 ± 11 kg/m^3^.

**Table 3:** Non-acoustic parameters of RGHS panels.

Grain size of rubber particles, (mm)	RG:HS ratio		ρ (true density), (kg/m^3^)		ρ (bulk density), (kg/m^3^)	τ_∞_	φ	Λ, (μm)	Λ', (μm)	σ, (kPa∙s/m^2^)
0.5–2		1:0		1293.73 ± 3.57	682.80 ± 31.62	2.17 ± 0.29	47.20 ± 2.38	228 ± 15	455 ± 29	20.2 ± 2.0
		9:1		1246.88 ± 5.25	616.55 ± 13.54	1.88 ± 0.15	50.03 ± 0.64	229 ± 9	457 ± 18	16.4 ± 1.0
		4:1		1168.72 ± 7.48	542.35 ± 18.50	2.18 ± 0.37	52.66 ± 1.26	245 ± 20	490 ± 41	15.6 ± 1.1
		2.3:1		1189.67 ± 6.97	564.56 ± 7.21	2.19 ± 0.17	52.93 ± 0.37	272 ± 11	545 ± 21	12.7 ± 0.8
		1.5:1		1194.65 ± 9.39	549.07 ± 4.00	2.12 ± 0.16	53.96 ± 0.36	254 ± 10	509 ± 20	13.8 ± 0.9
		1:1		1187.77 ± 5.14	509.87 ± 11.67	2.05 ± 0.12	56.80 ± 0.70	285 ± 9	571 ± 17	10.1 ± 0.6
2–4		1:0		1145.69 ± 3.23	571.52 ± 32.48	2.40 ± 0.08	48.15 ± 0.81	213 ± 90	560 ± 10	14.4 ± 2.6
		9:1		1129.55 ± 9.35	593.99 ± 19.03	2.79 ± 0.43	48.51 ± 0.46	279 ± 23	558 ± 46	14.7 ± 2.8
		4:1		1120.25 ± 10.33	526.75 ± 11.50	1.81 ± 0.09	52.57 ± 1.30	317 ± 8	633 ± 16	6.8 ± 0.6
		2.3:1		1096.88 ± 4.13	509.06 ± 25.96	1.73 ± 0.13	54.68 ± 1.29	302 ± 11	604 ± 22	7.9 ± 1.4
		1.5:1		1089.73 ± 9.97	506.03 ± 15.67	1.91 ± 0.09	54.71 ± 0.87	263 ± 112	626 ± 15	8.1 ± 2.0
		1:1		1073.61 ± 6.51	451.73 ± 26.61	2.23 ± 0.14	56.82 ± 1.37	380 ± 12	760 ± 23	6.2 ± 1.5
4–6		1:0		1148.4 ± 6.2	653.8 ± 18.4	1.89 ± 0.78	44.3 ± 0.8	294 ± 60	588 ± 119	10.8 ± 1.4
		9:1		1139.0 ± 4.6	566.0 ± 22.6	1.78 ± 0.29	50.3 ± 0.6	327 ± 26	653 ± 52	7.5 ± 0.7
		4:1		1123.2 ± 6.2	524.3 ± 26.8	1.93 ± 0.25	51.4 ± 1.4	350 ± 23	701 ± 45	6.9 ± 0.6
		2.3:1		1116.1 ± 1.0	562.3 ± 29.6	2.11 ± 0.01	52.7 ± 0.3	361 ± 1	722 ± 2	7.0 ± 0.4
		1.5:1		1063.1 ± 5.3	480.7 ± 18.2	2.09 ± 0.21	54.8 ± 0.8	430 ± 21	860 ± 41	4.7 ± 0.2
		1:1		1080.5 ± 7.8	473.6 ± 17.1	2.10 ± 0.15	56.9 ± 0.7	414 ± 15	828 ± 30	4.9 ± 0.2

The increasing ratio of hemp shives from 1:0 to 1:1 in a composite, results in the decrease of true density by 8% for rubber grain size 0.5–2 mm; for rubber grain size panels of 2–4 and 4–6 mm, true density decreased by 6%. Bulk densities showed the decrease as well: for rubber grain size from 0.5 to 2 mm bulk density decreased by 25%; for 2–4 mm rubber grain size by 21%; for 4–6 mm grain size 28%. The results obtained fit well within linear regression model, and the coefficient of determination *R*^*2*^ varied from 0.81 to 0.98. Furthermore, the same tendency was found with an air flow resistivity *σ*.

With the increase of the hemp shives the airflow resistivity for 0.5 – 2 mm grain size decreased by 50%, for 2–4 mm grain size – 57%, for 4–6 mm – 55%. Similarly, in terms of density, airflow resistivity showed strong correlation with the increase in the quantity of hemp shives, the coefficient of determination *R*^*2*^ varied from 0.70 to 0.89.

The correlation between the hemp quantity and high frequency tortuosity *τ*_*∞*_ was not found. Which means that hemp shives do not impact the pore path in the composite material.

The porosity of the material was measured directly. The results showed that the porosity values increased with the amount of hemp shives in the material, even though binder quantity was increased to compensate the absorbency of the hemp shives. The results showed a very strong linear correlation between the increase in the quantity of hemp shive and porosity. The porosity values of the RGHS panels of 0.5 – 2 mm grain size showed increment of 17%, of panels with 2–4 mm grain size 15%, 4–6 mm grain size 22%. The coefficient of determination *R*^*2*^ in all cases of the sizes of rubber grains in the panels varied from 0.92 to 0.94.

The empirically estimated viscous thermal length and characteristic thermal length also show moderate, strong or even very strong linear correlation with hemp quantity. The coefficient of determination *R*^*2*^ in all cases of the sizes of rubber grains in the panels ranged from 0.54 to 0.91.

The accuracy of the model does not show any evidence that it is related to a composition of the material. The calculated accuracy of the model ranged from 19.2 ± 5.3% to 31.6 ± 1.6%.

### SEM analysis of RGHS samples

**Fig. 10:**
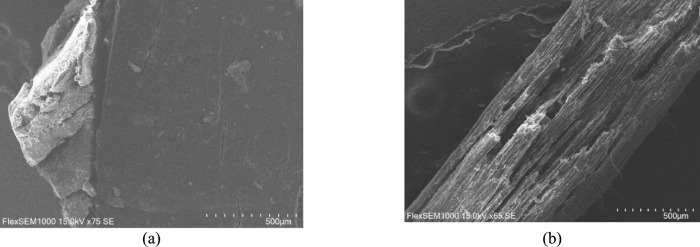
SEM micrographs of raw materials. **(a)** rubber granule, **(b)** hemp shive.

In this section SEM microscopy analysis is presented. Firstly, we analysed the pictures of raw rubber granules and hemp shive (Fig. 10).

In the Fig. 4 is shown raw rubber granule (Fig. 10 (a)) and hemp shive (Fig. 10 (b)) micrographs. The micrographs show that in both rubber and hemp shive particles has various gaps and cracks which suggests that it is suitable material for sound absorption applications.

The rubber granule exhibits an irregular and rough surface with visible cracks, pores, and fractured regions. Such a morphology indicates a high surface area. The porous and uneven surface also contributes to effective sound energy dissipation through internal friction and scattering mechanisms.

The hemp shive micrograph reveals a fibrous and elongated structure with numerous grooves, voids, and open capillaries along the fibre length. These features indicate a highly porous and anisotropic morphology, favourable for sound absorption.

**Fig. 11:**
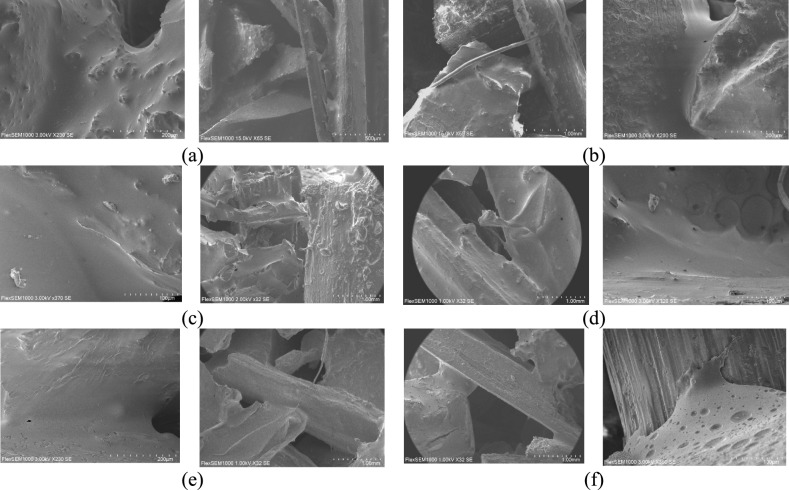
SEM micrographs of binder connections in composites with different hemp shive contents: **(a)** binder connections of the composite with rubber hemp ratio 9:1 (rubber grain size 0.5–2 mm), **(b)** binder connections of the composite with rubber hemp ratio 1:1 (rubber grain size 0.5–2 mm), **(c)** binder connections of the composite with rubber hemp ratio 9:1 (rubber grain size 2–4 mm), **(d)** binder connections of the composite with rubber hemp ratio 1:1 (rubber grain size 2–4 mm), **(e)** binder connections of the composite with rubber hemp ratio 9:1 (rubber grain size 4–6 mm), **(f)** binder connections of the composite with rubber hemp ratio 1:1 (rubber grain size 4–6 mm).

Figure 11 presents SEM micrographs of binder connections in composites with different RG and HS ratios and rubber grain size. In less zoomed pictures it is noticeable that the material has porous nature and can be used for sound absorption and thermal insulation applications.

In the RGHS composite samples with a 9:1 ratio (Fig. 11 (a), (c), and (e)), the polyurethane binder forms a relatively continuous film around the rubber particles, ensuring good encapsulation and interfacial adhesion. The resin fills the interparticle voids, providing structural cohesion and uniform bonding. In all cases, the polyurethane resin exhibits good wetting and an even distribution across the particle surfaces.

In the RGHS composite samples with a 1:1 ratio (Fig. 11(b), (d), and (f)), the polyurethane binder forms a slightly less continuous film around the rubber and hemp particles. Even though resin fills the interparticle voids, providing structural cohesion and uniform bonding, but there can be found some uncoated regions and occasional bubbles. Such phenomena also visible composites containing larger rubber granules (4 – 6 mm) (Fig. 11 (e) and (f)), where the degree of wetting is slightly reduced. Localized uncoated regions and occasional bubbles can be observed. The amount of binder was increased in these samples due to the presence of HS which absorbs part of the resin and affects the overall binder distribution. Even though the quantity of the binder was increased with the quantity of the hemp shives, the micrographs evident that connections are looser and there are more voids. Furthermore, such results correlates with porosity and flow resistivity results presented in Sect. 3.1.

When comparing samples by HS content – (a) versus (b), (c) versus (d), and (e) versus (f) –the binder wetting and surface coverage remain relatively similar, indicating that the presence of hemp shive does not significantly hinder resin spreading. However, in the 1:1 ratio composite, the binder tends to accumulate around the fibrous hemp surface, suggesting partial absorption into the fibre structure and enhanced mechanical interlocking at the interface.

### Sound absorption of RGHS panels

**Fig. 12:**
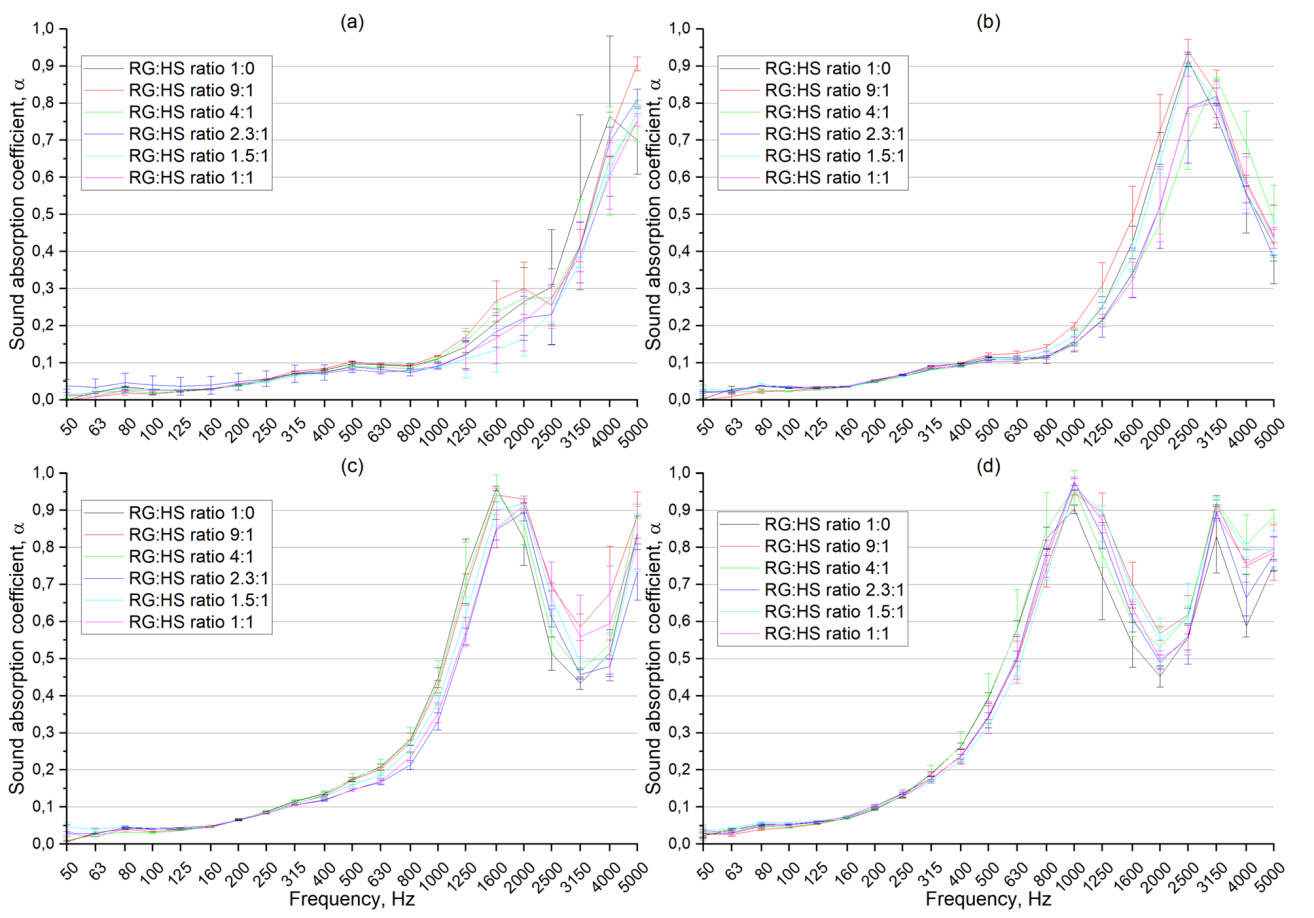
Sound absorption coefficient as function of frequency of 0.5–2 mm rubber grain size RGHS panels. **(a)** panel thickness 10 mm, **(b)** 20 mm, **(c)** 30 m, **(d)** 50 mm.

In this section, the results of the sound absorption coefficient (SAC) of the RGHS panels are presented. To determine the SAC, the transfer function method was used as described in Sect. 2.3. The sound absorption coefficient as a function of frequency of 0.5–2 mm rubber grain size RGHS panels presented in Fig. 12. The graphs are presented so that it would be easier to analyse the influence of hemp shives on SAC. First, it was clearly seen that the peak SAC values shift to lower frequency range as the thickness of the panels was increased. Such phenomena called ¼ wave length rule where maximum sound absorption occurs when the thickness of a porous material is approximately one-quarter of the wavelength of the sound it is intended to absorb, furthermore at this thickness, a pressure node and velocity antinode of the standing wave coincide at the surface of the material, allowing maximum energy transfer into the sound absorbing material and thus optimal absorption^57^.

Across all thickness levels, the quantity of hemp shives positively influences sound absorption, particularly at higher thicknesses; however, in most cases, the effect is within the range of the uncertainty of the measurements, especially in high-frequency range. Such results make the effect of hemp shives on sound absorption less acoustically significant, even though the effect is most notable in the mid- to high-frequency ranges, which are critical for practical acoustic applications. Although increasing the hemp content generally improves SAC, optimal performance does not strictly correlate with the highest hemp quantity; intermediate contents (2.3:1 and 1.5:1 ratios) frequently yield the best results. Error bars indicate measurement variability, with larger deviations occurring at higher frequencies. The binder increment to make solid panel as mentioned in method section, masked the possible double porosity effect of hemp shives in the material, therefore the changes in SAC are within error limits. The maximum peak sound absorption coefficient reaches 0.97 at 1 kHz when thickness of the sample was 50 mm.

**Fig. 13:**
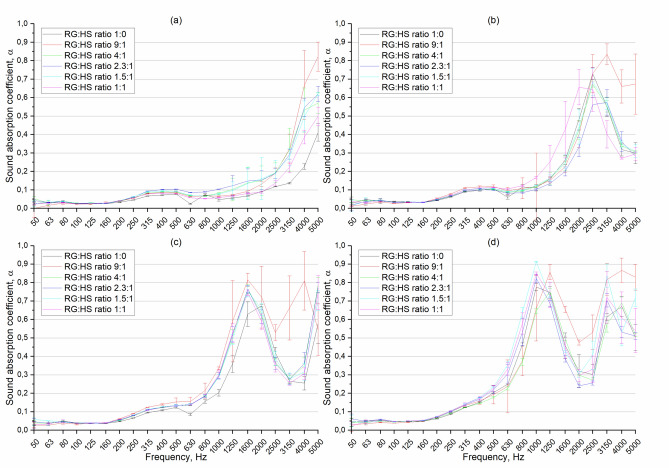
Sound absorption coefficient as function of frequency of 2–4 mm rubber grain size RGHS panels. **(a)** panel thickness 10 mm, **(b)** 20 mm, **(c)** 30 mm, **(d)** 50 mm.

The frequency-dependent SAC of 2-4 mm rubber grain size RGHS panels presented in Fig. 13. Same as shown in a description of results of panels of rubber grain size of 0.5-2 mm (Fig. 12) the SAC shifts to lower frequency range when thickness of the material is increased. Across all thicknesses, the use of grain rubber of 2–4 mm resulted in lower sound absorption coefficients compared to what is typically observed with finer particles (eg, 0.5–2 mm). In case of 2–4 mm rubber grain size RGHS composite panels, the variation with 9:1 RG to HS ratio stands out compared with other. Such results can be justified by the fact that this RGHS panel showed the highest flow resistivity values 14.7 ± 2.8 kPa∙s/m^2^ and similar porosity values (0.48) compared to the 1:0 RG:HS ratio panel. This combination of non-acoustic properties of the 9:1 RG:HS ratio panel led to higher SAC values. Although 9:1 ratio RGHS samples showed highest sound absorption values, but maximum peak SAC was found when measured 1.5:1 ratio RGHS panels. The peak SAC reached 0.91 at a frequency of 1 kHz.

**Fig. 14:**
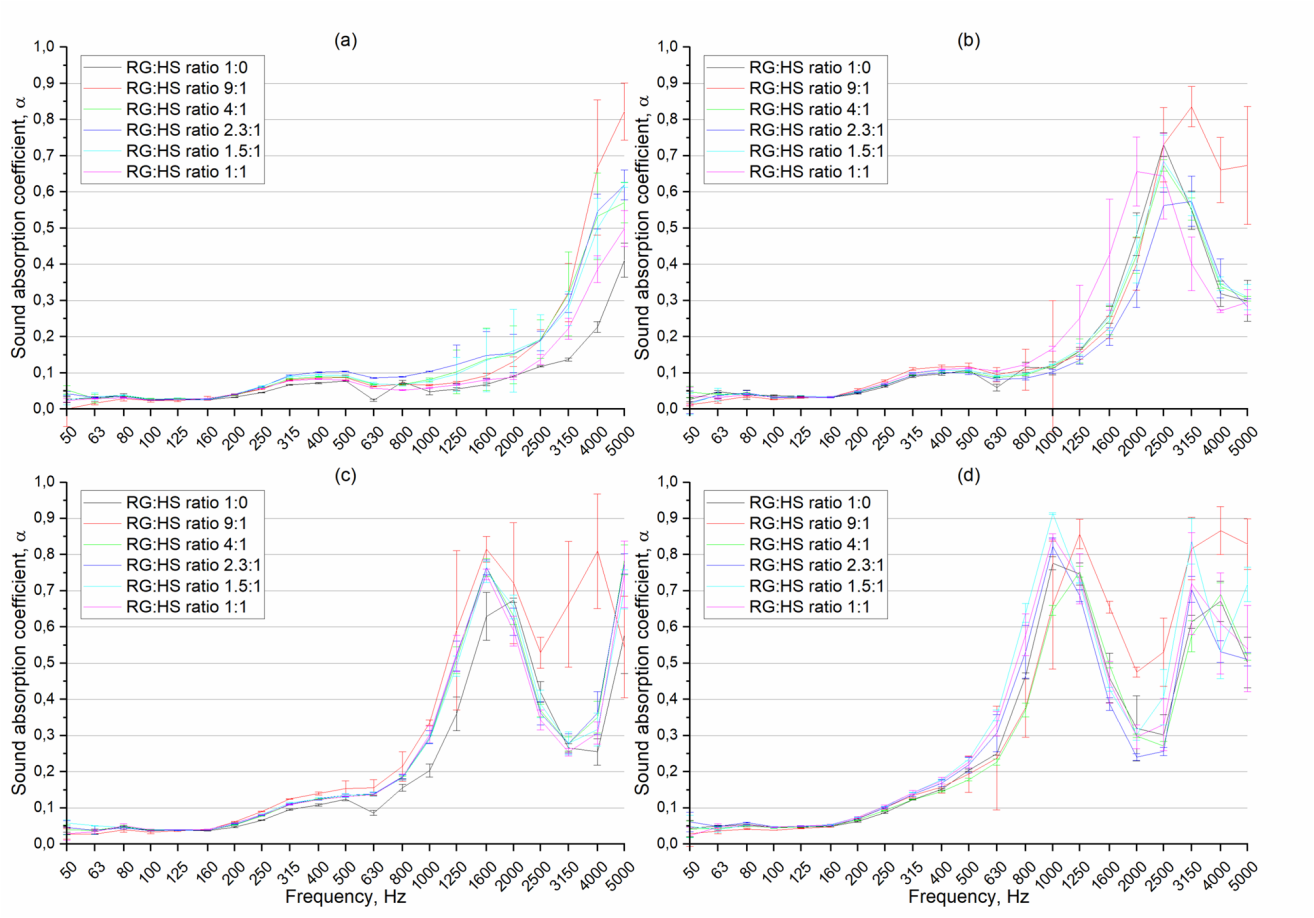
Sound absorption coefficient as function of frequency of 4–6 mm rubber grain size RGHS panels. **(a)** panel thickness 10 mm, **(b)** 20 mm, **(c)** 30 mm, **(d)** 50 mm.

The frequency-dependent SAC of 4–6 mm rubber grain size RGHS panels presented in Fig. 14. Similarly, to 0.5-2 mm rubber grain size panels, the amount of hemp shive does not influence the SAC significantly, especially in samples of 30 and 50 mm thickness. The SAC values of the 4–6 mm RGHS panels are slightly lower compared with 0.5–2 mm and 2–4 mm RGHS panels. The peak sound absorption value reached 0.91 at 1 kHz when the sample thickness was 50 mm. The highest sound absorption peak was found when hemp shives was not used. In general, it was observed that increasing the grain size of the rubber granules leads to the reduction of the SAC values. This result could be justified by the fact that increasing the size of rubber grain significantly reduced the airflow resistivity values. The average reduction in air flow resistivity when comparing the same ratio but different grain size panels was 30%.

### Sound absorption average results

**Fig. 15:**
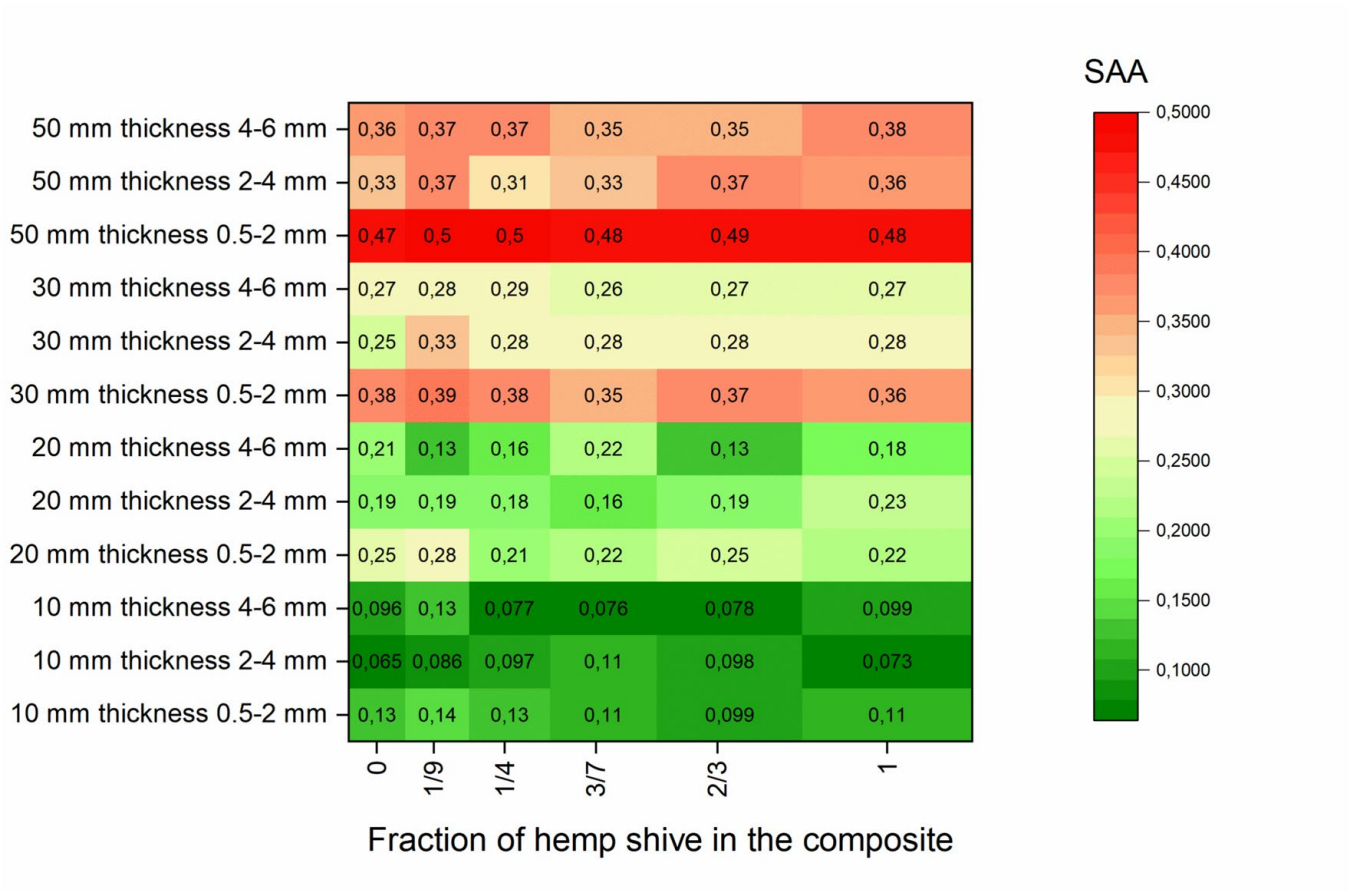
The heatmap of SAA as a function of quantity of hemp shives and particle size of rubber granules in RGHS composite panels.

In this section of the paper the SAA results are evaluated. In Fig. 15 the heatmap graph of SAA is presented. Heatmap graphs was generated using Origin 2019b Academic software^58^. The results indicate that the sample thickness has a significant positive effect on the sound absorption performance. In general, thicker samples exhibit higher SAA values across all hemp shive quantities. Specifically, samples with a thickness of 50 mm achieved the highest SAA values, reaching up to 0.50. On the contrary, thinner samples (10 mm) showed substantially lower sound absorption, with SAA values ranging between approximately 0.06 and 0.14.

The size of the rubber grain also markedly influences the SAA. Samples incorporating finer rubber granules (0.5–2 mm) consistently show higher SAA values compared to those containing coarser fractions (4–6 mm). This trend is especially pronounced in 50 mm thick samples, where the use of fine grains in combination with hemp shives leads to SAA values approaching 0.50. Coarser grain sizes showed lower SAA (0.31–0.38) values in all thicknesses, indicating that a denser and finer material structure promotes enhanced sound absorption.

Also, in most of the cases it is seen that the addition of HS to the composite material has very little or no influence on SAA rating at all. The difference of SAA values of the same-thickness RGHS panels varying in range from 0.03 to 0.1.

The optimal combination for relatively high SAA values is 50 mm thickness, fine rubber grain size (0.5–2 mm) and moderate hemp shive content ratio 9:1 and 4:1. Under these conditions, the material achieves the highest observed SAA values (0.50). On the contrary, samples with minimal thickness and coarse rubber granules demonstrate lower sound absorption performance.

To test what parameter (rubber grain size, hemp quantity and sample thickness) of the material are most influential, one-way and two-way ANOVA statistical analysis models were used. According to one-way ANOVA, the rubber grain size had a statistically significant effect on the sound absorption coefficient of the RGHS composites (value of F = 3.51, p = 0.035). The mean SAA decreased from 0.30 (0.5 to 2 mm) to 0.23 (4 to 6 mm), indicating that smaller rubber granules improve sound absorption. However, the relatively low R^2^ (0.092) suggests that other structural factors also contribute to acoustic performance.

The variation in the quantity of hemp shive did not have a significant effect on the sound absorption coefficient (F value = 0.043, p = 0.9998). The mean SAA remained similar (around 0.25) in all samples, showing that the addition of hemp shives does not influence the acoustic performance.

When testing the thickness parameter, one-way ANOVA revealed a significant effect on sound absorption (F value = 133.39, p = $2\bullet {10}^{-28}$). The mean sound absorption coefficient increased from 0.10 for samples of 10 mm to 0.40 for 50 mm samples, showing that increasing panel thickness substantially improves acoustic performance. The model explained 85.5% of the total variation (R^2^ = 0.85), which confirms thickness as the dominant parameter influencing sound absorption.

Two-way ANOVA revealed that both panel thickness and rubber grain size significantly affected the sound absorption coefficient of the samples (thickness: F value = 356.15, p < 0.001; grain size: F value = 57.78, p < 0.001). Increasing the panel thickness and decreasing the grain size both leads to higher sound absorption. The combined model was highly significant (F value = 236.79, p < 0.001), indicating that these two structural factors together explain most of the variation in acoustic performance.

### Results of thermal conductivity coefficient

**Fig. 16:**
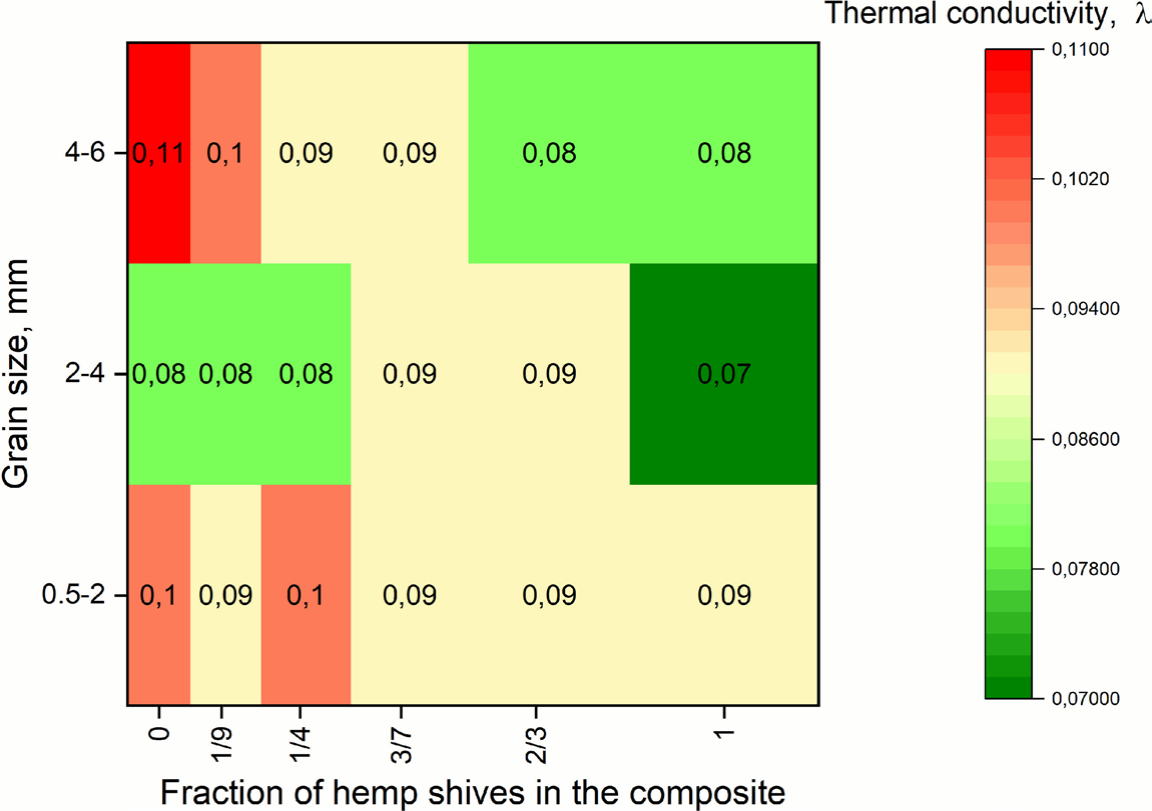
Thermal conductivity as function of quantity of hemp shives and rubber granule grain size in RGHS panels.

In this section, the results of thermal conductivity testing are presented. The Fig. 16 presents thermal conductivity (λ) values for RGHS panels that vary in grain sizes and different quantities of hemp shives. These values help to evaluate the thermal insulating performance of the proposed materials.

The data reveal that the size of the rubber grain has a more substantial impact on the thermal conductivity than the shive of hemp. Specifically, the coarsest rubber particles (4–6 mm) exhibit the highest λ values in all hemp contents, with a maximum of 0.11 W/m·K when the samples were without hemp shives. On the contrary, the intermediate grain size (2–4 mm) consistently shows the lowest thermal conductivity, dropping as low as 0.07 W/m·K at HS and RG ratio was 1:1.

One-way ANOVA showed a nearly significant effect of rubber grain size on thermal conductivity (F value = 3.26, p = 0.067). Although the correalization was not statistically significant and the p value was greater than 0.05, a decreasing trend was observed, suggesting that the size may slightly influence the thermal conductivity.

The effect of the hemp shive content on λ is not significant, but generally favourable to insulation performance. An increase in the hemp content tends to reduce the thermal conductivity or maintain it at a low level, particularly in samples with finer and intermediate rubber sizes. In the 2–4 mm grain size samples, λ remains at 0.08–0.09 W/m·K across most hemp contents and falls to 0.07 W/m·K at 1:1 RG and HS ratio. The 0.5–2 mm series remains relatively stable (0.09–0.10 W/m·K) regardless of the hemp content. One-way ANOVA revealed that hemp shive content did not influence the thermal conductivity performance of the composites (F value = 0.94, p = 0.489). The mean λ values varied minimally (0.086–0.097), suggesting that the addition of hemp does not notably affect thermal conductivity within the studied range.

Two-way ANOVA showed that rubber grain size had nearly significant influence on the λ parameter (F value = 3.64, p = 0.065), while hemp shive content showed no significant influence (F value) = 1.36, p = 0.318). The combined model was not significant as well (p = 0.153), suggesting that λ is only slightly dependent on grain size and unaffected by the addition of hemp in the composition.

This stability suggests that fine rubber particles help maintain a more consistent thermal performance, possibly due to a denser packing and reduced air pathways for heat transfer similarly to^59^. Meanwhile, the coarse particles contribute to slightly higher λ values, possibly due to greater internal porosity or less uniform material distribution.

The optimal thermal insulation performance was achieved using a 2–4 mm rubber grain size and hemp shive with ratio 1.5:1 and 1:1, where λ values were optimised (down to 0.07 W/m·K). This configuration is likely to provide the best combination of material structure and reduced thermal conductivity for insulating applications.

**Fig. 17:**
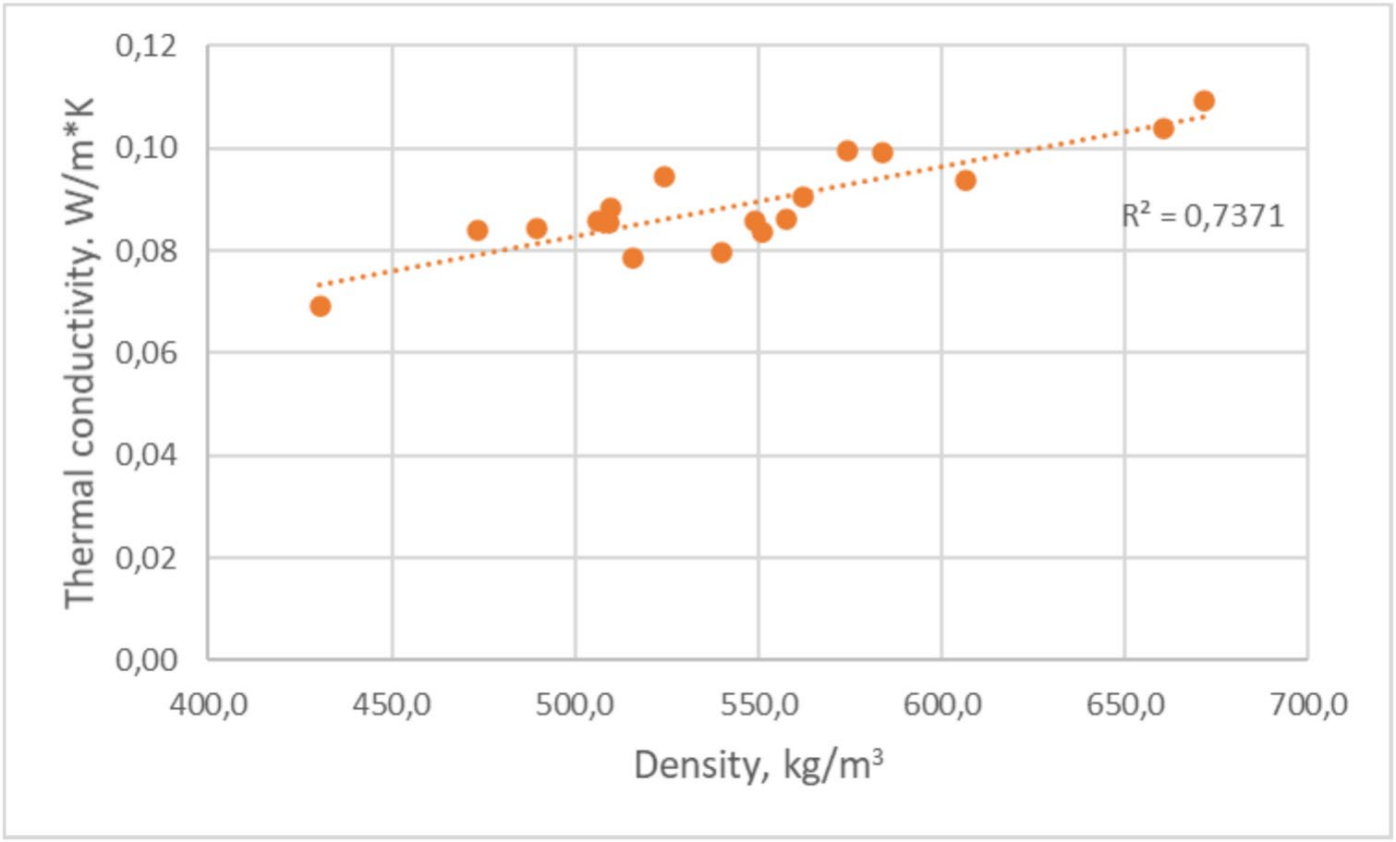
Dependence of thermal conductivity coefficient with RGHS panels density.

The analysis of thermal conductivity dependence form density (Fig. 17) reveals a noticeable relationship. Generally, an increase in density correlates with an increase in thermal conductivity, which is consistent with observations in other porous and composite materials. This pattern becomes evident when comparing samples with varying rubber granule sizes and different amounts of hemp shive (HS) content. The presented coefficient of determination *R*^*2*^ = *0.74,* which show strong the correlation between thermal conductivity and bulk density. The p-value is less than 0.05 which suggest that the regression model is statistically significant.

The lowest thermal conductivity value recorded was 0.07 W/m·K, found in a sample with rubber granule to hemp shive ratio 1:1 and intermediate-sized rubber granules (2–4 mm), with an approximate density of 430.5 kg/m^3^. In contrast, one of the highest thermal conductivity values, 0.104 W/m·K, occurred in a denser sample (671.6 kg/m^3^) made exclusively of small rubber granules (0.5–2.0 mm). This suggests that as the material becomes denser, the pathways for heat transfer through the solid phase increase, likely due to tighter particle compaction. As a result, thermal conductivity also increases. Lower-density samples, particularly those with a higher proportion of hemp shives, tend to exhibit lower thermal conductivity values, indicating better insulation performance. Meanwhile, the thermal conductivity values for fine rubber granule composites remain relatively stable in the range of 0.09 to 0.10 W/m·K, regardless of hemp content. This consistency may be attributed to improved packing density and reduced internal air voids, which limit heat transfer variability. Overall, the optimal thermal conductivity was observed in samples with intermediate rubber grain sizes combined with high hemp in ratios 1.5:1 and 1:1. These compositions achieved the most favourable balance between material structure and thermal performance. The findings align with other research on bio-based composites such as hempcrete, where lower densities are typically associated with superior insulating capabilities.

## Discussion

Recent studies have shown that composites made from recycled rubber or hemp shive composites exhibit high SAC values when optimised for porosity and structure. Rubber panels using fine granules (< 2 mm) and low binder content achieved maximum absorption coefficients of up to α ≈ 0.98 at mid-to-high frequencies^60^. Similarly, low-density hempcrete composites demonstrated high absorption, with peak values of α ≈ 0.90 around 700 Hz^61^. As shown in this study, parameters influencing SAC values include thickness, particle size, and binder type and content. Studies employing impedance tube methods (ISO 10534–2) confirm that sustainable rubber and hemp can match or exceed traditional acoustic materials, offering environmentally friendly alternatives for building and industrial applications^62–64^. RGHS panels showed similar peak SAC (0.91–0.97) but at slightly higher frequencies compared with results in other studies. The 60 mm thickness mineral wool SAC peaks to 0.85 at frequencies 2.5 kHz^65^, which is much higher frequency range compared with RGHS. The RGHS panels peaked at 1 kHz when the thickness was 50 mm. Such findings show a good correlation with existing studies, and we were able to show that rubber granule panel density could be reduced using hemp shives without sacrificing the sound absorption properties of the composite material.

RGHS could be considered as granular material. The grain size of the granular media dictates the density and porosity of the material. The granular media has unique property regarding the thermal conductivity. The thermal conductivity increases with density, due to higher particle packing and compression. Similarly, as in this study it was found in other studies that smaller particles leads to higher density values, thus increasing the thermal conductivity^11,66^.

Many studies report low thermal conductivity in biobased composites intended for building insulation. For example, hemp-lime blocks (hempcrete) (density ~ 300–400 kg/m^3^) have λ ≈ 0.04–0.06 W/m·K, making them effective insulators^67^. In the same study the authors stated that hempcrete porosity and compaction affect λ. Another study found that hemp shive with magnesium binder show λ increase of 0.062 W/m·K at 223 kg/m^3^ to 0.122 W/m·K at 483 kg/m^3^ (dry state) – higher density produces higher thermal conductivity^68^. The RGHS samples showed similar thermal conductivity values (0.07–0.11) compared to other similar materials prepared using hemp shives. Compared to traditional materials such as mineral wool (0.04 W/m·K) and polyurethane foams (0.045 W/m·K)^69,70^ RGHS panels showed slightly higher values. Such results mean that the rubber granule in the composite does not show a negative effect on the thermal insulation properties.

In this paper a composite material made of rubber granules and hemp shive bonded with recycled polyurethane resin was proposed. One of the objectives of this study was to use the rubber and hemp shive waste as it is prepared in factories without any pre-treatment to make the possible production of RGHS panels more feasible. Such a decision led to a limitation that made the RGHS panels vary in the microstructure between panels, and ultimately resulted in relatively high uncertainties of the tests. The grain size of the rubber and hemp shive could vary greatly due to quite high grain sieving size range, and the concentration of different grain sizes in the panel could vary within that range. The main positive aspect to take from the experiment data was that the bulk density of the panels decreased significantly without sacrificing efficiency. Such a result is extremely important for applications; therefore, lower density efficient materials are valued in the building construction sector.

Although the present study focused on the acoustic and thermal properties of the composites, the proposed panel preparation method is relatively simple and could be used in larger-scale production. The method requires only standard mixing and moulding equipment, suggesting good potential for industrial scalability. Since the raw materials of the panel are (recycled rubber granules and hemp shives) inexpensive and widely available, the overall production cost should be lower compared with conventional synthetic acoustic panels.

Such material could be used for sound absorption applications in noise barriers or filler in the walls as a sound absorbing layer. The innovation of this study lies in developing a composite material that combines recycled rubber granules and hemp shives bonded with regenerated polyurethane resin, creating a sustainable solution for acoustic panels. This material not only use waste components, reducing environmental impact, but also provides dual functionality—sound absorption and thermal insulation—making it highly suitable for building applications where energy efficiency and acoustic comfort are critical.

Future studies will address the issue with relatively high uncertainties in the tests. In the future, it is planned to test the mechanical properties of the RGHS and incorporate it in the noise barrier of which the sound-absorbing structure would be made of recycled materials.

## Conclusions

The results showed that RGHS composite panels could be used for sound absorption applications in the future. The peak sound absorption coefficient ranged from 0.60–0.97 depending on the thickness and composition. The highest SAA value (0.49) was reached with sample of 0.5–2 mm rubber grain size sample, rubber granule, and hemp shive ratio 1.5:1, thickness 50 mm. The changes in SAC were not significant since the increment of SAC was within the limits of the uncertainty of the measurements. Such results could be justified by the fact that hemp shives absorb the binder due to its fibrous structure, therefore the binder quantity was increased with hemp quantity to ensure good bonding of the particles. The provided SEM analysis showed that binder wetting in most of the samples were good and similar. The binder increment to make solid panel masked the possible double porosity effect hemp shives in the material. The ANOVA statistical analysis model showed that there was no statistical significance between hemp shive quantity and SAA values (p = 0.99), grain size showed marginal significance (p = 0.067), and sample thickness showed high significance (p < 0.001).

Non-acoustic parameters are influenced by the addition of hemp shives to the RGHS panels. First, changing rubber granule and hemp shive from 1:0 to 1:1 resulted in a decrease of airflow resistivity by 50–57% depending on the size of the rubber grain. Second, the test results showed that the true density decreased by 6 to 8%, the bulk density decreased by 21 to 28%, porosity increased by 12 to 17%, the characteristic and viscous thermal lengths increased by 20 to 29% and the high frequency tortuosity had no significant correlation with the quantity of hemp shives.

The addition of hemp shives showed improved thermal conductivity properties. Such results indicate that RGHS could be developed as multipurpose material for sound absorption and thermal insulation applications. The optimal result was 0.07 W/m·K which according to ISO 10456 falls into the category of wood wool boards and loose-fill expanded clay with loose fill. According to ANOVA, thermal conductivity was not significantly affected by hemp shive content (p = 0.489) or rubber grain size (p = 0.067).

The main findings of this study showed that proposed RGHS panels could be used as sound absorbing and thermal insulation material. The highest SAA values reached up to 0.49 and thermal conductivity as low as 0.07 W/m·K.

## Data Availability

The data that support the findings of this study are available from corresponding author but restrictions apply to the availability of these data, which were used under license for the current study, and so are not publicly available. Data are however available from the authors upon reasonable request and with permission of Innovation Agency Lithuania.
